# Knowledge, Attitudes, and Practices (KAP) Relating to Avian Influenza (H10N8) among Farmers’ Markets Workers in Nanchang, China

**DOI:** 10.1371/journal.pone.0127120

**Published:** 2015-05-18

**Authors:** Shengen Chen, Zifen Li, Maohong Hu, Shuangli Guo, Jingwen Wu, Bin Wang, Wei Hu, Yanshuang Sun, Hui Li, Mingbin Liu, Justin B. Moore, Haiying Chen

**Affiliations:** 1 Nanchang Centre for Disease Control and Prevention, Nanchang, Jiangxi Province, The People’s Republic of China; 2 School of Public Health, Medical College of Nanchang University, Nanchang, Jiangxi Province, The People’s Republic of China; 3 Department of Health Promotion, Education, and Behavior, Arnold School of Public Health, University of South Carolina, Columbia, South Carolina, United States of America; Linneaus University, SWEDEN

## Abstract

Three cases of avian influenza virus H10N8 were reported in Nanchang, China, as of April 2014. To identify the knowledge, attitudes, and practices (KAP) related to H10N8 among farmers’ market workers, a cross-sectional survey was conducted in 63 farmers’ markets in Nanchang. Using the resulting data, characteristics of poultry and non-poultry workers’ knowledge, attitudes, and practice were described. Results suggest that interventions targeting high-risk workers should be developed and implemented by public health agencies to prevent the spread of H10N8. Additionally policies that encourage farmers’ market workers to receive influenza vaccine should be developed, adopted, and enforced.

## Introduction

Based on the antigenic properties of the hemagglutinin (HA) and neuraminidase (NA) glycoproteins, influenza A viruses are categorized into 18 HA and 11 NA subtypes. All subtypes were identified initially from avian species, except for H17N10 and H18N11 subtype found in fruit bats [1, 2]. Among these subtypes, highly pathogenic avian influenza (HPAI) viruses are characterized by systemic infections, high mortality and morbidity, while low pathogenic avian influenza (LPAI) viruses usually cause asymptomatic infection or mild disease in poultry and wild birds. Occasionally, some HPAI and LPAI viruses, such as H5N1 and H7N9, can spread into humans and cause severe, sometimes fatal, disease, and pose a serious threat to the public’s health. The first case of human infection with avian influenza virus (AIV) (H5N1) was detected in Hong Kong in 1997 [3]. As of March 20, 2015, the WHO has reported 430 deaths in 16 countries attributable to H5N1, with a case fatality ratio of 55% [4]. In March 2013, human infection with the novel avian influenza A virus (H7N9) was detected in Shanghai, China [5]. Subsequently, the H7N9 virus spread to more than 10 provinces and municipalities, mainly in eastern China. Between April and June 2013, 5 cases of H7N9 were identified in Nanchang, China, in the Jiangxi province [6]. Fortunately, all of the infected individuals in Nanchang survived, but as of February 23, 2015, H7N9 has caused 571 laboratory confirmed cases and 212 deaths in China [7]. In December 2013, a new strain of AIV (H10N8) was isolated from a fatal case of severe pneumonia in Nanchang [8], which initiated emergency response and enhanced active surveillance for H10N8 virus in patients with pneumonia, and in live poultry markets. Three laboratory-confirmed cases of H10N8 in humans had been reported from urban parts of Nanchang as of April 2014. Although no direct epidemiologic link has been established between these cases, all of the cases had a history of visiting local live poultry markets before onset of the illness, which suggests the possibility of a linkage between infection and live poultry. Samples collected from live poultry markets were identified positive for novel H10N8 virus.

Previous studies have demonstrated that live poultry markets play an important role in AIV transmission from birds to humans [9–11]. Live poultry markets are high-risk locations for human infection with AIV [12]. Therefore, poultry workers, elderly consumers, or those with compromised immune systems visiting the live poultry markets might be at high risk of infection. Despite the utility of preventive behaviors to aid poultry workers and customers in avoiding infection during outbreaks of AIV, these preventive behaviors are adopted at low rates by the public, and these low adoption rates are influenced by their perceptions of the effectiveness of control measures [13], infectiousness and severity of the disease [14], and reliability of the information provided by public health authorities [15]. Thus, learning about the knowledge, attitudes, and behaviors of the public is crucial to improve communication efforts by public health officials.

We carried out a cross-sectional survey of knowledge, attitudes, and practices (KAP) among workers at farmers’ markets in Nanchang, China, to assess risk behaviors, preventive behaviors, and attitudes of the public in order to facilitate the development of effective prevention strategies against H10N8 infections.

## Methods

### Study sites and participants

The study was performed in 63 farmers’ markets in all 9 districts and counties of Nanchang, Jiangxi Province, China. Nearly 95% of the poultry sold in these markets originated from the same wholesale market, located in Qingshanhu District. Two categories of study participants, poultry workers and non-poultry workers, were recruited by convenience sampling from the 63 farmers’ markets. The poultry workers were defined as those involved in selling, slaughtering, plucking, cutting, or transporting poultry in the markets. The non-poultry workers included market managers, cleaners and those who sold vegetables, fish, meat or other foods in the markets. All subjects were local residents aged 17 to 75 years who provided written or verbal informed consent to participate in the investigation.

### Survey Methodology

The survey was conducted from December 29, 2013 through January 17, 2014, after the first case of H10N8 was reported. The investigators were local district or county Center for Disease Control and Prevention (CDC) staff members trained by officials from the Nanchang CDC. A self-designed, structured questionnaire was used to collect information on the general background of participants; knowledge, attitude, and practices (KAP) associated with avian influenza H10N8; and information sources on H10N8. Each interview lasted for approximately 15 minutes. Most questions were closed-ended: participants were instructed to choose from a pre-existing set of answers (Yes/No/Unknown). Most variables derived from these questions were categorical, with the exception of age, which was captured as a continuous variable. KAP associated with H10N8 were compared between poultry workers and non-poultry workers. Knowledge was assessed using 10 items that inquired about possible transmission routes and practices for prevention of H10N8, and the knowledge score was calculated by summing scores for correct answers. An eight-item practice assessment was used to derive a practice score that was calculated by summing the number of preventative measures the individuals reported regularly engaging in since receiving the news of H10N8 identification.

### Statistical Analysis

Data from the questionnaires were entered in duplicate and verified using Epi Data software (Odense, Denmark; available at http://www.epidata.dk/). Data were analyzed with SPSS (version 13.0; SPSS Inc., Chicago, IL, USA). Medians and Interquartile range (IQR) values were calculated for continuous variables, and were compared between poultry workers and non-poultry workers using the Mann-Whitney U test. For categorical variables, frequencies for poultry and non-poultry workers were compared using chi-square tests and Fisher’s exact tests. Knowledge questions were scored so that 3 points were assigned if the answer was ‘Yes’, 2 points were assigned for ‘unknown’, and 1 point was assigned for ‘No’. For the practice score, ‘Yes’ was coded 1, while “No” was coded 0. To standardize scores for comparability between knowledge and practice, the knowledge and practice scores were adjusted (i.e., knowledge score/3; practice score*1.25) to give a total score range of 0–10. Factors associated with participants’ knowledge and practice scores were analyzed using multiple linear regression models, employing a step-wise selection method to select participant characteristic variables for the final model. The errors are unobservable random variables, assumed to have zero mean and uncorrelated elements, each with common variance. If the errors are normally distributed, so are the residuals. The histogram and normal P-P plot of regression-standardized residual indicated that the distributions of the residuals from the models were approximately normal, and the Durbin-Watson test indicated that the residuals were independent. We used the Akaike Information Criterion (AIC) to select the best model. For all analyses, pairwise deletion was employed, thus participants with missing values were excluded on a test-by-test basis. Two-tailed test were utilized with a p-value < 0.05 considered significant.

### Ethics

The study protocol and informed consent procedure were approved by the Ethics Committee of Nanchang CDC. All study participants provided either written (if literate) or verbal (if illiterate) informed consent. Consent was documented with the participants’ signature or figure print if they were illiterate. Parent’s written informed consent was obtained first if the participant was under the age of 18.

## Results

### Demographics

A total of 887 workers agreed to participate in the present study, including 319 poultry workers (mean age 44.31±9.166, male 49.5%) and 568 non-poultry workers (mean age 46.87±10.133, male 40.8%). Most participants reported an elementary school or junior high school education level (37.1% and 39.2%, respectively). Education level was higher in the poultry workers, which included 57.3% with an educational background of junior high school or higher, compared with 47.9% in non-poultry workers (p = 0.002). A small percentage of poultry workers (8.9%) and non-poultry workers (8.2%) lived in the farmers’ market. A significant difference was observed in annual income levels (p < 0.01): 78.7% of poultry workers’ annual incomes were under 50,000 Yuan, compared to 88.0% in non-poultry workers (GDP per capita in Nanchang was 64,678 Yuan in 2013).

Thirty-five (11.0%) poultry workers reported that they had experienced contact with sick or dead poultry in the last month, and 25 (71.4%) of them had used protective measures, such as wearing mask, gloves, work clothes and other equipment. Four (0.7%) non-poultry workers reported that they had experienced contact with sick or dead poultry in the last month, and two of them had used protective measures. Only three poultry workers reported that they had received seasonal influenza vaccine in the past year (Table 1).

**Table 1:** Demography characteristics of farmers’ market workers in Nanchang, China.

Characteristic		Poultry worker (n = 319)	Non-poultry worker (n = 568)	p-value^*^	Total (%)
**Male (%)**		158(49.5)	232(40.8)	0.012	390(44.0)
**Age** (mean ± SD)		44.31±9.166	46.87±10.133	< 0.001	45.94±9.865
**Education** ^**#**^ (%)	Illiteracy	27(8.5)	75(13.4)	< 0.001	102(11.6)
	Elementary school	108(34.2)	217(38.8)		325(37.1)
	Junior high school	150(47.5)	193(34.5)		343(39.2)
	Senior high school	23(7.3)	60(10.7)		83(9.5)
	College and above	8(2.5)	15(2.7)		23 (2.6)
**Place of residence** ^**&**^ (%)	Living inside of the market	27(8.9)	43(8.2)	0.714	70 (8.4)
	Living outside of the market	276(91.1)	483(91.8)		759(91.6)
**Annual income, Yuan** ^‡^ (%)	< = 30,000	103(33.1)	316(57.5)	< 0.001	419(48.7)
	30,001–50,000	142(45.6)	168(30.5)		310(36.0)
	50,001–100,000	58(18.6)	62(11.3)		120(13.9)
	> 100,000	8(2.6)	4(0.7)		12(1.4)
**Contact with sick or dead poultry in the past month** (%)		35(11.0)	4(0.7)	< 0.001	39(4.4)
**Adopted protective measures after contacting with sick or dead poultry**		25(71.4)	2(50.0)	-	27(69.2)
**Influenza vaccination in the last year**		3(0.9)	0	0.011**	3(0.3)

^**#**^11,

^**&**^58,

^‡^26 records were excluded from analysis due to existence of missing values.

^*^ Chi-square tests (Age: T-tests).

^**^Fisher’s exact tests.

### Information source and their association with behaviors related to H10N8

Awareness of H10N8 was low in both poultry workers (57.7%) and non-poultry workers (60.2%, p = 0.011). Television was the most common information source (56.6%), followed by newspapers (25.5%), friends (10.7%), and the internet (5.7%). The majority (61.9%) of participants said they did not worry about being infected with H10N8, while 31.0% of participants reported worry about H10N8 infection. Regarding participants’ opinions on H10N8 information published by the government, 95.0% of participants thought the government issued the information in a timely manner, with only 7.7% of poultry workers and 3.6% of non-poultry workers reporting that the information was not timely. Twenty-four percent of participants were suspicious of government reports of H10N8. The percentages of participants who took additional individual precautions following the news of the H10N8 outbreak by wearing mask, gloves and overalls, and washing hands increased (27.2%, 24.3%, 49.6% and 70.9%, respectively). Only 38.5% participants reported regularly washing their hands with water and soap or hand sanitizer. Most participants (70.3%) ventilated their living quarters more frequently than before the news of the H10N8 outbreak, but a lower proportion of participants disinfected their stalls (22.8%). Poultry workers took more protective measures than non-poultry workers (practice scores 4.43 vs. 3.60, p < 0.001), such as wearing a mask (33.2% vs. 24.0%, p = 0.018); wearing overalls (55.4% vs. 46.5%, p = 0.037); or sanitizing stalls (31.0% vs. 18.4%. p = 0.001) (Table 2 and Table 3).

**Table 2:** Information sources and reactions of farmers’ market workers after occurrence of H10N8 infection in humans in Nanchang, China.

Characteristic		Poultry worker (n = 319)	Non-poultry worker (n = 568)	p-value*	Total (%)
**Having heard about H10N8**		184(57.7)	342(60.2)	0.470	526(59.3)
**H10N8 Information source**	Television	112(60.9)	185(54.3)	0.171	297(56.6)
	Newspaper	39(21.2)	95(27.9)		134(25.5)
	Internet	14(7.6)	16(4.7)		30(5.7)
	Friend	16(8.7)	41(7.2)		57(10.9)
	Others	3(1.6)	4(0.7)		7(1.3)
**Worry about being infected with H10N8** ^**#**^	Don’t worry	206(66.7)	326(59.3)	0.072	532(61.9)
	Worry	81(26.2)	185(33.6)		266(31.0)
	Very worry	22(7.1)	39(7.1)		61(7.1)
**How do you think about the information of H10N8 released by the government?** ^**&**^	Credible	146(79.8)	249(73.9)	0.003**	395(76.0)
	Partly credible, partly concealed	29(15.8)	85(25.2)		114(21.9)
	Less credible	7(3.8)	2(0.6)		9(1.7)
	Totally incredible	1(0.5)	1(0.3)		2(0.4)
**Do you think the government issued the information of H10N8 timely or not?** ^**&**^	Very timely	99(54.7)	177(52.2)	0.122**	276(53.1)
	Timely	68(37.6)	150(44.2)		218(41.9)
	Not timely	4(2.2)	3(0.9)		7(1.3)
	It doesn’t matter	10(5.5)	9(2.7)		19(3.7)
**Protective measures adopted**	Wearing mask	61(33.2)	82(24.0)	0.018	143(27.2)
	Wearing gloves	46(25.0)	82(24.0)	0.745	128(24.3)
	Wearing overalls	102(55.4)	159(46.5)	0.037	261(49.6)
	Washing hands	139(75.5)	234(68.4)	0.038	373(70.9)
	Washing hands without soap	82(60.7)	140(61.9)	0.820	222(61.5)
	Washing hands with soap or hand sanitizer	53(39.3)	86(38.1)		139(38.5)
	Sanitizing stall	57(31.0)	63(18.4)	0.001	120(22.8)
	Increasing the frequency of disinfection	36(19.6)	53(15.5)	0.244	89(16.9)
	Ventilation	141(76.6)	229(67.0)	0.032	370(70.3)
	Covering nose and mouth with a handkerchief when sneeze	58(31.5)	83(24.3)	0.084	141(26.8)

^**#**^28,

^**&**^6 records were excluded from analysis due to existence of missing values.

* Chi-square tests.

**Fisher’s exact tests.

**Table 3:** Knowledge and practice scores in farmers’ market workers in Nanchang, China.

Characteristic		Mean	Median	IQR	Z*	p-value
**Knowledge score**	Poultry worker(N = 317)	8.07±1.492	8.33	6.67–9.33	-3.967	< 0.001
	Non-poultry worker(N = 560)	8.52±1.218	9.00	7.67–9.67		
**Practice score**	Poultry worker(N = 319)	4.43±2.862	3.75	2.50–6.25	-4.324	< 0.001
	Non-poultry worker(N = 568)	3.60±2.781	3.75	1.25–5.00		

*The Z statistic was obtained from the Mann-Whitney test for two independent samples.

IQR, interquartile range.

### H10N8 KAP

KAP related to AIV are summarized in Table 4. Regarding the preventive measures, approximately four-fifths of participants believed that avoiding contact with poultry, wearing personal protection when in contact with poultry, washing hands with water and soap or sanitizer after touching poultry, and physical exercise can prevent infection with AIV (79.6%, 78.8%, 83.2% and 82.6%, respectively). Regarding the risk factors for infection with H10N8, the greatest proportion of participants believed that touching sick or dead poultry may cause infection (76.6%), followed by slaughtering or processing (58.6%), feeding (57.7%), and transporting live poultry (55.6%). These values were significantly higher in non-poultry workers than in poultry workers (79.4% vs. 71.5%, 62.5% vs. 51.7%, 62.9% vs. 48.6% and 59.3% vs. 48.9%, respectively). There were also 36.2% participants who believed that selling frozen poultry products might cause infection with H10N8. Only one-fifth of participants believed that frequent visits to farmers’ market might cause infection with H10N8. The non-poultry workers displayed higher AIV knowledge scores than the poultry workers (8.52 vs. 8.07, p < 0.001) (Table 3).

**Table 4:** Knowledge-attitude related to H10N8 in farmers’ market workers in Nanchang, China.

Characteristic		Poultry worker (n = 319)	Non-poultry worker (n = 568)	p-value*	Total (%)
**Which measures can prevent you from infecting with H10N8?** ^*^	Avoiding contact with poultry	221(69.3)	485(85.4)	< 0.001	706(79.6)
	Taking personal protective equipments when contact with poultry	248(77.7)	451(79.4)	0.506	699(78.8)
	Washing hands with soap or sanitizer after touching poultry	256(80.3)	482(84.9)	0.141	738(83.2)
	Physical exercise	258(80.9)	475(83.6)	0.436	733(82.6)
**Which exposure may lead to infection with H10N8?** *	Touching sick or dead poultry	228(71.5)	451(79.4)	0.001	679(76.6)
	Feeding live poultry	155(48.6)	357(62.9)	< 0.001	512(57.7)
	Transporting live poultry	156(48.9)	337(59.3)	0.002	493(55.6)
	Slaughtering or processing live poultry	165(51.7)	355(62.5)	0.002	520(58.6)
	Selling frozen poultry products	106(33.2)	215(37.9)	0.029	321(36.2)
	Often go to the farmers' market	65(20.4)	126(22.21)	0.244	191(21.5)
**What will you do if you are sick with fever, sneeze, and cough?** ^**#**^	Purchasing cold medicine by myself	97(30.7)	225(40.0)	0.045	322(36.7)
	Seeking medical service from private clinic	119(37.7)	190(33.8)		309(35.2)
	Seeking medical service from hospital	60(19.08.8)	84(14.9)		144(16.4)
	With none treatment	40(12.7)	63(11.2)		103(11.7)
**Do you support the following H10N8 control measures implemented in farmers’ markets?** ^**&**^	Closure and sanitization of markets	43(13.6)	100(17.9)	0.409	143(16.3)
	Sanitization but not closure of markets	213(67.2)	355(63.5)		568(64.8)
	Neither closure nor sanitization of markets	25(7.9)	40(7.2)		65(7.4)
	Slaughtering all poultry in markets	36(11.4)	64(11.4)		100(11.4)
**Do you believe that keeping good hand hygiene could prevent infection with H10N8?** *		270(84.6)	512(90.1)	0.019	782(88.2)
**Do you believe that sanitization of markets regularly could prevent infection with H10N8?** *		259(81.2)	456(80.3)	0.236	715(80.6)

^**#**^9,

^**&**^11 records were excluded from analysis due to existence of missing values.

*Numbers of the participants whose answer was Yes.

**Chi-square tests.

Over 80% participants of both groups thought that keeping good hand hygiene and sanitizing farmers’ markets regularly could prevent H10N8 infection. Nearly 31% of poultry workers and 40% of non-poultry workers said they would rather use home remedies than seek medical advice when they felt sick, but 12.7% of poultry workers and 11.2% of non-poultry workers believed that they would recover from AIV infection without any treatment. Over 60% of participants in both groups indicated support for the item ‘sanitize often, but do not close the market’. Nearly 7.5% of participants in the poultry and non-poultry workers supported ‘neither close nor sanitize the markets’ (Table 4).

### Multiple regression analysis

Factors associated with participants’ KAP scores are summarized in Table 5 and Table 6. Briefly, age, education level, place of residence, occupation, annual income and concern about infection with H10N8 were significantly associated with knowledge score (Table 5). The participants who live outside of the market or were a non-poultry worker had lower practice scores (Unstandardized coefficients -1.505 and -0.896, respectively). The participants who were worried, or very worried about infection with H10N8 had taken more protective measures than those who did not worry (Unstandardized coefficients 0.556, and 1.222, respectively).

**Table 5:** Multiple regression analysis for possible influencing factors of knowledge scores among farmers’ market workers in Nanchang, China.

Factors		Coefficients*	Standard error	t	p-value
**Constant**		6.454	0.424	15.218	< 0.001
**Age**	17–29^**#**^	-	-	-	-
	30–39	-0.541	0.223	-2.428	0.015
	40–49	-0.510	0.210	-2.427	0.015
	50–59	-0.305	0.218	-1.402	0.161
	> = 60	-0.532	0.260	-2.048	0.041
**Education level**	Illiteracy^**#**^	-	-	-	-
	Elementary school	0.310	0.153	2.024	0.043
	Junior high school	0.556	1.159	3.506	< 0.001
	Senior high school	0.508	0.208	2.450	0.015
	College and above	0.898	0.319	2.817	0.005
**Place of resident**	Live in the market^**#**^	-	-	-	-
	Live outside of the market	0.488	0.163	2.984	0.003
**Occupation**	Poultry worker^**#**^	-	-	-	-
	Non-poultry worker	0.510	0.098	5.225	< 0.001
**Annual Income, Yuan**	< = 30,000^**#**^	-	-	-	-
	30,001–50,000	0.146	0.105	1.396	0.163
	50,001–100,000	0.061	0.140	0.437	0.662
	> 100,000	-0.849	0.395	-2.148	0.032
**Worry about be infected with H10N8**	Don’t worry^**#**^	-	-	-	-
	Worry	0.325	0.101	3.208	0.001
	Very worry	0.622	0.179	3.480	0.001

^**#**^Reference group.

*Unstandardized coefficients.

**Table 6:** Multiple regression analysis for possible influencing factors of practice scores among farmers’ market workers in Nanchang, China.

Factors		Coefficients*	Standard error	t	p-value
**Constant**		8.024	0.747	10.74	< 0.001
**Place of resident**	Live in the market^**#**^	-	-	-	-
	Live outside of the market	-1.505	0.348	-4.321	< 0.001
**Occupation**	Poultry worker^**#**^	-	-	-	-
	Non-poultry worker	-0.896	0.202	-4.448	< 0.001
**Worry about be infected with H10N8**	Don’t worry^**#**^	-	-	-	-
	Worry	0.556	0.215	2.591	0.010
	Very worry	1.222	0.381	3.210	0.001

^**#**^Reference group.

*Unstandardized coefficients.

## Discussion

To date, three patients have been confirmed to be infected with H10N8 in Nanchang, China. All of them had visited live poultry markets before onset of disease. Studies examining human infections with AIV indicate that visiting live poultry market is a potential risk factor [7, 16]. The farmers’ market workers are regarded as a high-risk population for infection with AIV. However, our results suggest that the awareness of human infection with H10N8 is low in both poultry and non-poultry farmers’ market workers. Television was the main information source for H10N8 information in both groups, similar to the results found in studies of H5N1 KAP in Thailand and Vietnam [17, 18]. More than one in ten (11.0%) poultry workers reported an instance of contact with sick or dead poultry, compared with 0.7% of non-poultry workers, indicating that the poultry workers were more frequently exposed to the potentially contaminated animals. Studies have demonstrated that direct contact with sick or diseased poultry is the major risk factor for human infections with H5N1 [19]. Our study showed that more participants in both groups reported strengthening individual precautions following the H10N8 discovery by washing hands, but, only 36.5% of them washed hands with soap or hand sanitizer. Grayson and colleagues demonstrated that washing hands with soap and water is the most effective intervention in reducing influenza A virus infection in humans [20]. We also found low reported rates of protective measures, such as wearing a mask, wearing gloves, or sanitizing stalls. In contrast, these measures were relatively common in Italian poultry workers, as 87.9% washed hands, 59.9% wore gloves and 59.9% wore protective masks [21]. Generally, preventive measures are more common in those with greater knowledge, but that was not the case in the current study. In the current sample, the poultry workers reported more protective measures, but their knowledge scores were lower than the non-poultry workers. This may be because of the different occupation exposure, as the non-poultry workers may think that wearing a mask and gloves is unnecessary, especially those individuals working as a market manager or a vegetable vendor. In our multivariable analyses, H10N8 knowledge was predicted by education level, place of resident, occupation, and concern about infection with H10N8. Similar to a previous study, we found that the level of education was an important predictor of AIV knowledge [18], despite the low levels of education in the present sample. In addition, the results indicate that participants who lived outside the market had higher knowledge scores but lower practice scores compared to those who lived in the market. However, due to the cross-sectional nature of our study, our data cannot be used to determine whether increases in the practice of preventive measures among those living in the market resulted from increased awareness. The relatively low levels of preventive practices may be attributable to the low perceived threat reported by the workers, evidenced by the majority of participants (61.9%) reporting lack of worry about infection with H10N8. This lack of concern could be potentially due to the low number of individuals affected by the most recent outbreak. An encouraging finding was that the participants who worried about H10N8 infection displayed higher knowledge and practice scores than those who did not. These results suggest that government needs to strengthen health education messages, with targeting to high-risk populations (such as farmers’ market workers) emphasizing susceptibility to infection and increased performance of health protective behaviors.

Efficacious and safe vaccines remain the cornerstone of influenza prophylaxis in most countries [22]. In 2014, it was reported that the influenza vaccination rate in China was between 2% and 3% [23], with the highest rates in urban areas. For example, the 2009 the rate of influenza vaccination in Beijing was close to those observed in developed countries [24]. However, the influenza vaccination rates in our participants recruited from Nanchang were considerably lower than Beijing (i.e., less than 1%). As indicated in Li’s 2014 study, worry about infection was found to be the strongest predictor of vaccination uptake [25]. As such, the low level of concern regarding AIV infection expressed by workers is cause for alarm. The results indicate that staff of farmers’ markets did not attach importance to prevention and treatment of AIV. This attitude can potentially delay diagnosis and initiation of antiviral administration, and is not conductive to the prevention and control of human acquisition of AIV. As a result, improving access and utilization of healthcare by farmers’ market workers should be a target of public health agencies in China.

A study conducted in Hong Kong [26] showed that closing the live poultry markets two times a month could effectively reduce the rate of AIV infection. At present, the management measures implemented in Nanchang’s live poultry markets were to clean and disinfect daily, and close one day per month. In our study, only 13.6% of participating poultry workers and 17.9% of non-poultry workers supported the strategy of closure and disinfection of the market. Support for regularly disinfecting, but not closing, the market was 67.2% and 63.5% in poultry and non-poultry workers, respectively. The main reason was that market closure may seriously reduce the poultry workers’ income [27], thus financial compensation or reducing market stall rent may help poultry workers accept closure strategies more readily.

The results of the present study should be considered in light of a number of limitations. First, our questionnaire did not query participants for their reasoning behind their AIV related attitudes. For example, questions like ‘why don’t you worry about infection with H10N8’ or ‘why don’t you receive the influenza vaccination’, which could elucidate the possible relationship between AI attitudes, were missing. Additionally, no objective information, such as direct observation, was collected to confirm the self-reported behaviors. As such, the responses may have been influenced by social desirably. These limitations could be addressed in future investigations.

## Conclusions

The occurrence of H10N8 had not caused public panic yet, but the KAP of H10N8 in farmers’ market workers was not optimal. Interventions, potentially targeting high-risk workers, should be developed and implemented by public health agencies to prevent the spread of H10N8. Additionally, policies that encourage farmers’ market workers to receive influenza vaccine should be developed, adopted, and enforced. Hopefully, the present findings will provide a better understanding of influenza risk communication and education needs of farmers’ market workers in Nanchang and other underdeveloped cities in China.

## Supporting Information

S1 Dataset KAP associated with avian influenza H10N8 on farmers’ market workers gathered from 29 December 2013 to 17 January 2014. This is the original database that matched by the questionnaire.
(XLS)

S1 Questionnaire KAP associated with avian influenza H10N8 on farmers’ market workers. A self-designed, structured questionnaire was used to collect information on the general background of participants; knowledge, attitude, and practices (KAP) associated with avian influenza H10N8.
(DOC)
