# Supplementary material for: Knowledge, Attitudes, and Practices (KAP) Relating to Avian Influenza (H10N8) among Farmers’ Markets Workers in Nanchang, China
Source: PLoS One. 2015 May 18;10(5):e0127120. doi: 10.1371/journal.pone.0127120 (PMC4436285; doi:10.1371/journal.pone.0127120)
Supplement: S1 Questionnaire — A self-designed, structured questionnaire was used to collect information on the general background of participants; knowledge, attitude, and practices (KAP) associated with avian influenza H10N8. (DOC) [file pone.0127120.s002.doc]

**Form ID:**

**Date:**

Q1.Gender：□1.Male □2.Female

Q2.What is your age： years old

Q3. Nationality：□1.Ethic Han □2.Other (Specify______ ) □Unclear Q4. What is your highest education level completed?

□1.Illiteracy □2.Elementary school □3.Junior high school

□4.Senior high school □5.College/University and above

Q5. Where do you live？ □1.In the market □2.Outside of the market

Q6. What is your type of work in the farmers’ market?  □1.Poultry（□Sell， □Slaughter， □Transport） □2.Birds □3.Flowers and plants □4.Vegetable □5.Meat □6.Fish □7.Dry goods □8.sundry goods

□9.Market management □10.Pet (Specify______ )

Q7. **What is your annual income?**

□1.Under 30,000￥ □ 2. 30,000-50,000￥

□ 3. 50,000-100,000￥ □ 4. Above 100,000￥

**Q8. Have you touched sick or dead poultry by hands in the last month?**

□ 1.Yes（answer 8.1） □.2. No

**Q8.1Whether adopted preventive measure?** □1.Yes □2.No

Q9. **Whether ever heard about AI (H10N8)?** □ 1.Yes（answer 9.1） □ 2.No

**Q9.1 What’ s your information source？**

□1.Television □2.Newspaper □3.Internet □4.Friend □5.Other

**Q10. Are you worried about being infected with H10N8?**

□1.No worried □2.Worried □3.Very worried

Q11. Which measure can prevent infection of AIV?

Q11.1 Avoid contact with poultry ( )

□1.Yes □2.No □3.Unknown

Q11.2 Make personal protection when contact with poultry

□1.Yes □2.No □3.Unknown

Q11.3 After touching poultry, wash hands with soap or sanitizer

□1.Yes □2.No □3.Unknown

Q11.4 Exercise

□1.Yes □2.No □3.Unknown

Q12. Which measure causes infection of AIV?

Q12.1 Touching sick or dead poultry

□1.Yes □2.No □3.Unknown

Q12.2 Feed of live poultry（ ）

□1.Yes □2.No □3.Unknown

Q12.3 Transport of live poultry

□1.Yes □2.No □3.Unknown

Q12.4 Slaughter and processing of live poultry

□1.Yes □2.No □3.Unknown

Q12.5 Sales of frozen poultry products

□1.Yes □2.No □3.Unknown

Q12.6 Often go to the market

□1.Yes □2.No □3.Unknown

**Q13. After emergence of H10N8, what protective measures have you strengthen?**

Q13.1 Wear mask  □1.Yes □2.No

Q13.2 Wear gloves □1.Yes □2.No

Q13.3 Wear overalls □1.Yes □2.No

Q13.4 Washing hands □1.Yes □2.No

Q13.4.1 How do you wash your hands?

□ 1.Carefully without soap □2.use soap or hand sanitizer

Q13.5 Sanitize stall with disinfectant □1.Yes □2.No

Q13.6 Increase the frequency of disinfection □1.Yes □2.No

Q13.7 Ventilate □1.Yes □2.No

Q13.8 Use a handkerchief when sneeze □1.Yes □2.No

**Q14. What will you do if you are sick with fever, sneeze, cough？**

□ 1.Take the cold medicine by self □2.Go to the clinic

□ 3.Go to the hospital □ 4.No treatment

**Q15. Do you believe that the government published H10N8 information true**？

□ 1. Credible □ 2.Partly credible, partly concealed

□ 3.Less credible □4.Totally incredible

**Q16. Do you believe that the government published H10N8 information timely？**

□1.Very timely □ 2.Timely □3.Not timely □4.Not to matter

**Q17. Do you believe that keep a good hand washing can prevent infection with avian influenza** **virus?**

□1.Yes □2.No □3.Unknown

**Q18. Do you believe that disinfect the market can prevent infection with avian influenza virus？**

□1.Yes □2.No □3.Unknown

**Q19. Which measure do you support for the prevention and control of avian influenza in farmers’ market?**

□1.Close and sanitize the market □ 2.Not close but often sanitize the market

□ 3. Neither closes nor sanitizes the market □ 4.Slaughter

**Q20. Have you injected flu vaccine in the last years?**

□1.Yes □2.No □3.Unclear

**Investigator:**
